# Low-quality birds do not display high-quality signals: The cysteine-pheomelanin mechanism of honesty

**DOI:** 10.1111/evo.12549

**Published:** 2014-12-04

**Authors:** Ismael Galván, Kazumasa Wakamatsu, Pablo R Camarero, Rafael Mateo, Carlos Alonso-Alvarez

**Affiliations:** 1 Departamento de Ecología Evolutiva, Estación Biológica de Doñana – CSICc/ Américo Vespucio s/n, 41092, Sevilla, Spain; 3 Department of Chemistry, Fujita Health University School of Health SciencesToyoake, Aichi, 470-1192, Japan; 4 Instituto de Investigación en Recursos Cinegéticos (IREC) – CSIC-UCLM-JCCMRonda de Toledo s/n, 13005, Ciudad Real, Spain

**Keywords:** Genotypic quality, handicaps, house sparrow, melanins, signaling

## Abstract

The mechanisms that make that the costs of producing high-quality signals are unaffordable to low-quality signalers are a current issue in animal communication. The size of the melanin-based bib of male house sparrows *Passer domesticus* honestly signals quality. We induced the development of new bibs while treating males with buthionine-sulfoximine (BSO), a substance that depletes the levels of the antioxidant glutathione (GSH) and the amino acid cysteine, two elements that switch melanogenesis from eumelanin to pheomelanin. Final bib size is negatively related to pheomelanin levels in the bib feathers. BSO reduced cysteine and GSH levels in all birds, but improved phenotypes (bibs larger than controls) were only expressed by high-quality birds (BSO birds with largest bibs initially). Negative associations between final bib size and cysteine levels in erythrocytes, and between pheomelanin and cysteine levels, were observed in high-quality birds only. These findings suggest that a mechanism uncoupling pheomelanin and cysteine levels may have evolved in low-quality birds to avoid producing bibs of size not corresponding to their quality and greater relative costs. Indeed, greater oxidative stress in cells was not observed in low-quality birds. This may represent the first mechanism maintaining signal honesty without producing greater relative costs on low-quality signalers.

Signals are traits that convey information and evolve because of the benefits bestowed by the individuals to which this information is sent, that is, the signals' recipients. This represents the basis of biological communication (Hasson 1997; Bradbury and Vehrencamp 2011). Most biological signals are honest (Searcy and Nowicki 2005), as they influence the subsequent decisions of the signals' recipients in a way that, on average, the fitness of recipients is improved, as opposed to cheating signals, that decrease their fitness (Hasson 1994). Signal honesty can be achieved by signal design (e.g., amplifiers and indices), by convention (e.g., symbols and icons) and, lastly, by trade-off relationships between costs and benefits of signaling (i.e., handicaps; Hasson 1997). By far, the latter category has gathered most research on biological signaling, and some authors have even equaled honest signaling to handicaps (e.g., Searcy and Nowicki 2005). The handicap principle (Zahavi 1975) is therefore a cornerstone for the concept of honest signaling. It states that a high signal expression is limited to high-quality signalers, that is, those individuals bearing the specific genotypic quality or phenotypic condition that is sought by the signals' recipients (Hasson 1997). This is because only high-quality individuals can afford the costs derived from signal production or maintenance, so that low-quality signalers obtain lower fitness benefits from the recipients' decisions in proportion to the negative effects on fitness derived from signal costs (i.e., pay greater relative costs; Grafen 1990) or are less efficient at converting signaling into fitness (Getty 2006) for a given signal expression.

The nature of the costs of producing handicaps has been the focus of most research on animal signaling, aiming at answering the question of why only high-quality individuals display large signals (e.g., Folstad and Karter 1992; Olson and Owens 1998; Bird et al. 2001; McGraw 2008). Even if low-quality individuals face greater relative costs for producing a handicap signal, the possibility still exists that they may afford these costs and produce signals as large as those produced by high-quality individuals, for example, as an attempt to maximize residual fitness during terminal reproductive investment (Hasson 1994). In this case, handicaps would become dishonest, that is, bluffs (Hasson 1994). However, bluffs seem to be rare in nature or only observed in particular tactic strategies that report short-term benefits to signalers (Brown et al. 2012). It is assumed that the avoidance of dishonesty is mediated by the costs of producing handicap signals, which would be so large that they are prohibitively expensive for low-quality signalers (Maynard Smith and Harper 2003). This assumption, however, have several problems from both theoretical and empirical perspectives, as the evolution of signals may not be favored when it implies incurring at substantive costs, and such costs are not always found by empirical studies (Zollman et al. 2013). It has been suggested that identifying the mechanisms that prevent low-quality signalers from sending the costly signal is more useful to understand how signal honesty is maintained than studying the nature of the costs on honest signalers (Lachmann et al. 2001; Számadó 2011; Holman 2012). The mechanisms that prevent dishonesty in costly signaling are therefore not clear.

Evolutionary constraints may be an alternative explanation to production and maintenance costs, as some signals (e.g., traits that convey information on body size) are honest because their genetic basis makes them impossible to fake (Maynard Smith and Harper 2003; Holman et al. 2013). However, such traits should actually be signals that are honest by design (i.e., amplifiers and indices) but not handicaps, which indeed could be theoretically faked (Hasson 1997; Harper 2006; Vanhooydonck et al. 2007). The proximate mechanisms that prevent low-quality individuals from producing costly handicap signals is thus an open field that remains largely unexplored. This field is expected to provide responses that are crucial for understanding why most biological signaling systems seem to be honest (Searcy and Nowicki 2005) despite their theoretical susceptibility to be invaded by cheats (Johnstone and Grafen 1993).

In this study, we experimentally test the capacity of handicap signals generated by some pigments to explain the proximate mechanisms of dishonesty avoidance through the physiological pathway of melanin synthesis. Melanins are the most extended biological pigments, found in virtually all organisms (Hill 1992). Although the structure of melanins is heterogeneous, two main chemical distinct forms are identified in vertebrate animals: eumelanin, a polymer of 5,6-dihydroxyindole and 5,6-dihydroxyindole-2-carboxilic acid units, and pheomelanin, a polymer of benzothiazine and benzothiazole derivatives (Ito et al. 2011). Eumelanin mostly produce dark black color, whereas pheomelanin is responsible for reddish-chesnut traits. Animal synthesize eumelanin when the amino acid cysteine is depleted or in low levels in melanocytes, and pheomelanin when cysteine concentration is higher than a threshold level, although both pigments are usually produced at different proportions by the same cells (García-Borrón and Olivares Sánchez 2011; Riley et al. 2011). The main physiological reservoir of cysteine is the tripeptide glutathione (GSH), which is also the most important intracellular antioxidant (Wu et al. 2004). Therefore, pheomelanogenesis requires a constant supply of cysteine via GSH, which represents a consumption of this important antioxidant (Pavel et al. 2011; Galván et al. 2012a; Morgan et al. 2013).

By coloring their integument by producing pheomelanin or eumelanin under different conditions of oxidative stress (i.e., the imbalance between the production of reactive oxygen species and the state of the antioxidant and repair machinery), animals can thus signal their genotypic quality to conspecifics, as only those individuals with a high antioxidant capacity may be able to generate large melanic traits (Galván and Solano 2009; Galván and Alonso-Alvarez 2009; Hõrak et al. 2010). Indeed, many vertebrate species use melanic traits to honestly signal their quality to conspecifics (McGraw 2008), which permits high-quality individuals to obtain mating advantages or benefits during social interactions which ultimately can result in greater fitness outcomes (see Hõrak et al. 2010 and Kekäläinen et al. 2010 for examples of eumelanin-based signals; and Safran and McGraw 2004 and Clough et al. 2009 for example of pheomelanin-based signals). The biochemistry of melanogenesis therefore constitutes an appropriate theoretical background for understanding the evolution of signal honesty.

The black chest bib of male house sparrows *Passer domesticus* is a melanin-based plumage patch that constitutes one of the most intensively studied animal signals (Anderson 2006). Male house sparrows displaying larger bibs are dominant in aggressive interactions over other males, have better body condition and, in some populations, achieve higher lifetime reproductive success (reviewed in Nakagawa et al. 2007). Bib size therefore positively affects fitness and reflects overall genotypic quality in male house sparrows. This has been explained through the handicap principle, that is, low-quality house sparrows do not display large bibs because their production represents costs that are unaffordable by them (Jawor and Breitwisch 2003). In particular, immunocompetence has been considered as the factor mediating the production costs of large bibs, as bib expression depends on testosterone levels which in turn suppress the immune system, and immunosuppression costs would be only affordable by high-quality males (i.e., the immunocompetence handicap hypothesis sensu Folstad and Karter 1992; see González et al. 1999; Buchanan et al. 2003; Laucht et al. 2011; Laucht and Dale 2012). Some authors have directly manipulated the bib size of male house sparrows, showing that birds with experimentally increased bibs face a reduction in reproductive success (Veiga 1993) but not increased rates of aggressive interactions with other males or physiological stress levels (González et al. 2002). This suggests that, although cheaters (i.e., low-quality birds with experimentally increased bibs) may ultimately reduce their fitness, there are no costs preventing the maintenance of large bibs to low-quality birds. Males with large bibs have higher levels of testosterone-mediated immunosuppression (Møller et al. 1996; González et al. 1999; Evans et al. 2000), but to our knowledge there is no empirical demonstration that the physiological costs derived from immunosuppression are so high that the production of large bibs is not possible for low-quality birds (i.e., those with smaller bibs). Therefore, production costs are not enough to explain why the signaling system of house sparrows is not invaded by cheaters producing bluffs.

Here, we postulate that the evolutionary control of the honesty of house sparrows' bib can only be properly understood by exploring and manipulating the physiological mechanism that directly controls the integrative constituents of the bib, that is, melanins. With this aim, we induced the development of new bibs in male house sparrows during a period in which they were kept in captivity and some birds were treated with DL-buthionine-(*S,R*)-sulfoximine (BSO) whereas others served as controls. BSO is a specific inhibitor of γ-glutamylcysteine synthetase, the enzyme that catalyzes the rate-limiting step in GSH synthesis in which two of its three constitutive amino acids (glutamate and cysteine) are bonded, and thus decreases GSH levels with no side, toxic effects (Griffith 1982; Dizdar et al. 1997; Galván and Alonso-Alvarez 2008). BSO also increases cysteine catabolism, hence decreasing its levels (Griffith 1982; Dizdar et al. 1997). Because of these effects and because it reacts directly with the intermediate oxidation products of the melanogenesis pathway, BSO affects melanin production resulting in more soluble and degradable pigments, which should decrease the levels of both pheomelanin and eumelanin with a more marked effect on the latter (Galván et al. 2014b). In male house sparrows, bib size is negatively related to pheomelanin levels in the bib feathers (Galván et al. 2014a). The BSO treatment should thus induce the development of larger bibs.

We measured bib size in male house sparrows before (at capture, initial bib size) and after the experimental treatment (final bib size), considering initial bib size a measure of intrinsic overall quality (Møller 1992). If it is production costs what prevents low-quality birds from producing large bibs, as assumed by the handicap principle, the experimental induction of larger bibs should affect to both high- and low-quality birds but the latter should suffer from greater physiological costs. We investigated these costs by measuring oxidative stress in cells, as these should be the costs derived from the direct mechanism that produces larger bibs (i.e., decrease in cysteine and GSH levels, and thus, antioxidant capacity). We also investigated possible effects of treatment on the total antioxidant capacity (TAC) of plasma to explore the possibility that decreases in intracellular antioxidant levels are compensated by increases in the levels of circulating antioxidants (i.e., Galván and Alonso-Alvarez 2008; Galván et al. 2010), and on the body condition of birds. On the contrary, if BSO directly promotes the development of larger bibs and any physiological mechanism has evolved to avoid dishonesty in signal size, we predicted that the experimental induction of larger bibs would be only observed in high-quality birds (i.e., those with larger initial bibs), but blocked in low-quality birds.

## Materials and Methods

### EXPERIMENTAL DESIGN

In June–July 2010, 53 free ranging adult male house sparrows were captured with mist nests in the surroundings of *Dehesa Galiana* experimental facility (Ciudad Real, Spain) and housed alone in individual cages (0.6 m long × 0.4 m wide × 0.4 m tall; Italgabbie, Caltrano, Italy) in an indoor aviary (7.4 × 3.3 × 2.5 m). Light was provided by seven fluorescent lamps (120 cm, 40 W, Hg-A 1638) that resemble sun light with a constant regime of 16h:8h, L:D. The birds were provided with ad libitum water and food consisting of a commercial mixture of seeds for canaries (Kiki, Callosa de Segura, Spain) and cuttlefish bone to ensure the coverage of calcium needs. After capture, the birds were left for acclimation in the cages during one week.

**Figure 1:**
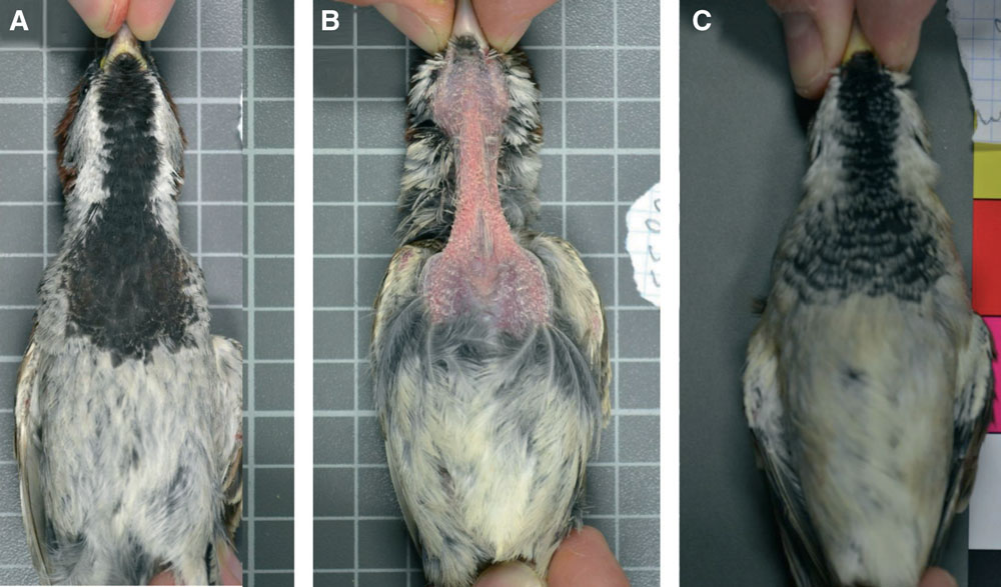
Male house sparrows used in the experiment showing (A) the initial bib just before the beginning of the experiment, (B) the bib after all its feathers and neighboring feathers were plucked in the beginning of the experiment, and (C) the final bib with newly grown feathers at the end of the experiment (taken from Galván et al. 2014a). Note in (A) that, although the predominant color of the bib is that conferred by eumelanin (i.e., black), the coexistence of pheomelanin is also visually appreciated by the presence of some orange feathers at the bottom of the plumage patch.

On 13 July, blood samples were taken with a syringe from the jugular vein of birds. A maximum of 200 μl of blood volume was taken following Diehl et al. (2001). The blood was immediately stored at 4°C and maintained for a maximum of 6 h until centrifugation at 4°C and 3500 × *g* during 5 min. After centrifugation, the plasma was separated from the cell portion and both parts were stored at −80°C until biochemical analyses were made. The same day, a photograph of the bib of birds was taken with a digital camera (Olympus E-50). The birds were held in the same posture, at a fixed distance from the camera, under standardized illumination (Fig. 1). Bib size was measured by selecting the total black area on throat and chest with Adobe Photoshop and converting pixels to cm^2^ (McGraw et al. 2003). The analysis of bib size was made by a technician blinded to the aims of the study. In the house sparrow, the molting period takes place at the time our experiment was conducted (i.e., July–September; Anderson 2006), but to avoid interindividual differences in the phenology of plumage molt, the day after samples were taken (14 July) we plucked the feathers of the bib patch (Fig. 1B), thus inducing the growth of all the bib feathers during the experiment (McGraw 2007). Bib feathers were plucked the same day that photographs to measure initial bib size were taken (13 July). To allow individuals to grow bib patches larger than they previously displayed if they are capable (i.e., if induced by the experimental treatment), the neighboring feathers that surround the black bib patch were also plucked at this time (McGraw 2007). Tarsus length and body mass measurements were taken with digital calipers and balance, respectively. All measurements taken before the beginning of the experiment on 13–14 July were referred to as “initial values.”

The experimental treatment started one week after the bib feathers were plucked (20 July). The birds were randomly assigned to one of two groups (BSO, 27 birds, or control, 26 birds). BSO was administered dissolved in the drinking water at a concentration of 5 mM, as this value can deplete GSH levels without causing toxicity in different tissues when provided in the drinking water to mice, which are similar to house sparrows regarding body mass (ca. 25–30 g; Watanabe et al. 2003). Control birds received water without BSO. Water suppliers were checked two days per week and new water was provided when water levels were one-fourth the length of suppliers or less.

The duration of the experimental treatment was 45 days. Just after the end of the treatment (3 September), the birds had developed most of the bib feathers, but to ensure that the length of all feathers was fully developed we provided all birds with water without BSO for another 24 days. On 27 September, the birds were captured and the final bib size was measured as explained above for initial bib size (Fig. 1C). Blood samples and body mass measurements were also taken the same day. All measurements taken after the end of the experiment on 27 September were referred to as “final values.” The birds were released back to nature at the same site where they were captured after all final measurements were performed.

### MEASUREMENT OF CYSTEINE LEVELS IN ERYTHROCYTES

We measured cysteine levels following the method developed by Švagera et al. (2012) for plasma. To apply this method to the analysis of erythrocytes, we added a first step consisting of a dilution of erythrocytes to 1:10 with a carbonate-buffered saline (5 mM Na_2_CO_3_ in saline) to induce cell lysis and thus facilitate the extraction of intracellular cysteine. The samples were analyzed by gas chromatography (GC) coupled to an electronic impact-mass spectrometry detector. The chromatographic system consisted of a 6890N Network GC System with a 5973 Network Mass Selective Detector (Agilent Technologies, Santa Clara, CA).

Mass spectra of the derivatized cysteine and an internal standard (*p*-chlorophenylalanine, PCP) were obtained in a continuous scanning mode from 50 to 450 *m/z*, using for quantification *m/z* ions 220 and 210 for cysteine and PCP, respectively (retention time—cysteine: 2.70, PCP: 3.38). The ions used for identification were 220, 102, 74, and 204 for cysteine and 210, 102, 125, and 212 for PCP. Cysteine levels are expressed as micromoles per gram of pellet. The repeatability of this technique based on 17 erythrocyte samples from which two aliquots were separately analyzed was high (*r* = 0.86, *P* < 0.0001).

### MEASUREMENT OF GSH LEVELS IN ERYTHROCYTES

Total GSH (tGSH) levels in erythrocytes were determined by following the method described by Tietze (1969) and Griffith (1980) with some particular modifications. Details of the use of this technique with bird samples are published elsewhere (Galván and Alonso-Alvarez 2008). High repeatability values of this method have been published elsewhere (e.g., Alonso-Alvarez et al. 2010). Concentration is presented as micromoles of GSH per gram of pellet.

To determine oxidized GSH (GSSG) levels, 8 μl of 2-vinylpyridine were added to an aliquot (400 μl) of the supernatant obtained for tGSH assessment to promote GSH derivatization. The mixture was then centrifuged (3500 × *g* for 10 min), and the change in absorbance of the supernatant was assessed at 405 nm. High repeatability values of this method have been published elsewhere (e.g., Alonso-Alvarez et al. 2010). Reduced GSH levels were calculated by subtracting GSSG levels to tGSH levels. The ratio GSH/GSSG was used as an index of oxidative stress in cells.

### MEASUREMENT OF URIC ACID LEVELS AND ANTIOXIDANT CAPACITY IN PLASMA

Uric acid concentration of plasma was assessed in 5 μl of plasma using a Bio-Tek microplate reader (PowerWave XS2, Bio-Tek Instruments Inc., Winooski VT) fixed at 520 nm. The uricase/peroxidase method was used (kits from Biosystems, Barcelona, Spain). Repeatabilities in 45 samples assessed twice was very high (*r* = 0.99, *P* < 0.001). TAC of plasma was determined through a colorimetric assay. This method is based upon the color change, adapted from Erel (2004), caused by the addition of hydrogen peroxide to colorless 2,2V-azinobis(3-ethylbenzo-thiazoline-6-sulfonate), which oxidizes it into a characteristic blue-green solution. Repeatabilities in plasma samples assessed twice in other passerine (zebra finch *Taeniopygia guttata*) gave a high repeatability (*r* = 0.86, *P* < 0.0001, *N* = 394; Romero-Haro and Alonso-Alvarez 2014).

### MEASUREMENT OF MALONDIALDEHYDE LEVELS IN PLASMA

The protocol of Agarwal and Chase (2002) with modifications by Nussey et al. (2009) was followed to quantify malondialdehyde (MDA) in plasma in several laboratory sessions. Samples and standards were injected into an Agilent 1100 Series HPLC system (Agilent, Waldbronn, Germany) fitted with a fluorescence detector set and a 5 μm ODS-2 C-18 4.0 × 250 mm column maintained at 37°C. The mobile phase was MeOH:KH2PO4 (50 mM; 40:60 v/v), running isocratically for 10 min at a flow rate of 1 mL min^−1^. Data were collected at 515 nm (excitation) and 553 nm (emission). Although we did not estimate the repeatability of this assay in house sparrows, repeatabilities calculated from plasma samples assessed twice in other passerine species (zebra finch) were very high (intra- and intersession: *r* > 0.97, *N* = 20, *P* < 0.001; Romero-Haro and Alonso-Alvarez 2014).

### MEASUREMENT OF MELANIN LEVELS IN FEATHERS

The analysis of melanins was based on the formation and detection by HPLC of specific degradation products, 4-amino-3-hydroxyphenylalanine (4-AHP) by reductive hydrolysis of pheomelanin with hydriodic acid and pyrrole-2,3,5-tricarboxylic acid (PTCA) and thiazole-2,4,5-tricarboxylic acid (TTCA) by alkaline H_2_O_2_ oxidation of eumelanin and pheomelanin, respectively. Thus, 4-AHP and TTCA are specific to pheomelanin and PTCA is specific to eumelanin. Details of sample analyses have been published elsewhere (Galván et al. 2012b).

### STATISTICAL ANALYSES

As bib size reflects genotypic quality in male house sparrows (reviewed in Nakagawa et al. 2007), we divided birds into two groups regarding their “intrinsic” quality: those with initial bib size equal or lower than the median initial bib size of all birds were considered as “low quality,” while those with initial bib size larger than the median initial bib size were considered as “high quality” (see Galván and Sanz 2008 for a similar statistical treatment of birds regarding overall quality). This index of quality was homogeneously distributed among experimental treatments (generalized linear model with quality as binomial response variable and treatment (BSO vs. control) as a fixed factor: 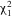 = 0.17, *P* = 0.676). There were 25 low-quality birds (12 controls and 13 treated) and 26 high-quality birds (14 controls and 12 treated; low plumage uniformity prevented us from determining quality in two birds). Some variables measured in blood could not be taken in some initial samples due to hemolysis, so that initial sample is lower than final sample size in some comparisons.

General linear models (GLMs) were used to analyze the association between final bib size and cysteine and GSH final levels. Variation in body size was controlled for by adding tarsus length as a covariate. As birds at the end of the experiment had been exposed to the experimental treatments, final bib size may be affected by this potential source of variation, so treatment (control vs. BSO) was added to the model as a fixed factor. As birds of different quality may respond differently to the different treatments (see predictions for the study in the Introduction), bird quality was added as another fixed factor. We also included the interactions between cysteine or GSH levels and quality, between cysteine or GSH levels and treatment, and between treatment and quality. There was a clear tendency of final levels of cysteine and GSH to be negatively correlated (*r* = −0.25, *N* = 46, *P* = 0.090), so cysteine and GSH were separately considered in different GLMs.

To analyze the effects of treatment and quality on the change in the levels of cysteine and GSH during the course of the experiment, we used GLMs including the time at which measurements were taken (initial or final) as a repeated-measures (i.e., within-subjects) effect. Treatment, quality (low-quality vs. high-quality) and their interaction were added as fixed factors. In the sake of simplicity, we only show the results of the effects of the interaction between the within-subjects factor and the other predictors (i.e., all effects in these models refer to the interaction between the within-subjects factor and the predictor of interest). There were no differences between control and BSO-treated birds in the initial levels of neither cysteine (one-way ANOVA: *F*_1,48_ = 0.89, *P* = 0.350) nor GSH (*F*_1,44_ = 0.38, *P* = 0.543).

Similar repeated-measures GLMs were used to analyze the effects of treatment on the production of melanins, with initial and final melanin levels as response variables. To estimate the total melanin content in the bib, we multiplied the melanin concentration by bib size, assuming that melanin content is homogeneously distributed along the bib. Treatment, quality, and their interaction were added to the models as fixed factors to explore the possibility that the effect of treatment differs between low- and high-quality birds, while tarsus length was a covariate to control for individual variation in body size. Before analyzing the effects of treatment on the production of melanins, we analyzed the association between the levels of melanins and the levels of cysteine and GSH, as melanin production should depend on the latter. With this aim, we used GLMs considering the final levels of melanins, cysteine, and GSH. Given the negative association between cysteine and GSH levels (see above), cysteine and GSH were separately considered in different GLMs. There were no differences between control and BSO-treated birds in the initial melanin levels (4-AHP: *F*_1,38_ = 0.04, *P* = 0.846; TTCA: *F*_1,38_ = 0.57, *P* = 0.455; PTCA: *F*_1,38_ = 0.02, *P* = 0.894).

Similar repeated-measures GLMs were also used to analyze the effects of treatment and quality on change in TAC, ratio GSH/GSSG, MDA, body condition (i.e., size-independent body mass) and bib size, including treatment, quality, and their interaction as fixed factors. In the model for TAC, we also controlled by the change in uric acid levels (e.g., Galván et al. 2010) by adding initial and final levels of this variable as a changing covariate. In the model for body condition, initial and final body mass were response variables and tarsus length a covariate. In the model for bib size, tarsus length was a covariate to control for body size. There were no differences between control and BSO-treated birds in the initial levels of TAC (*F*_1,49_ = 1.88, *P* = 0.177), GSH/GSSG (*F*_1,42_ = 0.07, *P* = 0.794), MDA (*F*_1,49_ = 2.04, *P* = 0.160), body condition (*F*_1,51_ = 0.03, *P* = 0.857), or bib size (*F*_1,47_ = 0.85, *P* = 0.362), nor between low- and high-quality birds in the initial levels of body condition (*F*_1,48_ = 0.21, *P* = 0.650).

In all models, a backwards stepwise procedure was used to remove nonsignificant terms, using a *P*-value of 0.1 as a threshold to abandon the model. Inspections of residuals confirmed that the normality assumption was fulfilled. When interactions between two factors were significant, differences between factor levels were analyzed by means of Fisher least significant difference (LSD) post hoc tests. All statistical analyses were made with Statistica 8.0 (StatSoft, Tulsa, OK) excepting models including changing covariates, which were run with Statistica 5.0.

## Results

### RELATIONSHIP BETWEEN FINAL BIB SIZE AND CYSTEINE AND GSH LEVELS

**Figure 2:**
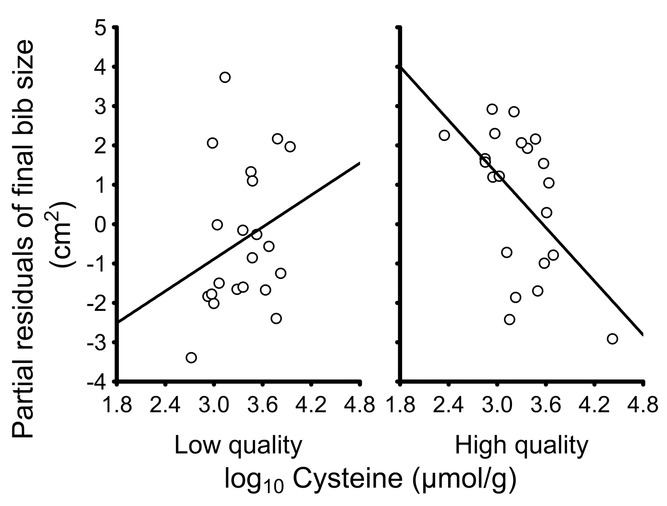
Relationship between final bib size and cysteine levels in erythrocytes of male house sparrows of low and high quality. The residual figures of the response variable (i.e., partial effects after applying a GLM model without cysteine levels and quality) are shown. The line is the regression line.

There was a significant interaction between cysteine levels and quality (*F*_1,35_ = 4.16, *P* = 0.049; cysteine: *F*_1,35_ = 0.43, *P* = 0.516; quality: *F*_1,35_ = 5.19, *P* = 0.029) that arose as a consequence of a significant negative correlation between final bib size and cysteine levels in high-quality birds (*b* = −1.83, *t* = −2.13, *P* = 0.040) and a lack of correlation in low-quality birds (*b* = 0.96, *t* = 0.92, *P* = 0.363; Fig. 2). The final model also included a significant interaction between treatment and quality (*F*_1,35_ = 8.71, *P* = 0.006; treatment: *F*_1,35_ = 0.95, *P* = 0.336; tarsus length: *F*_1,35_ = 0.01, *P* = 0.905), but the effect of this interaction on bib size is analyzed in detail in the last section below.

Another model showed a similar significant interaction between GSH levels and quality (*F*_1,35_ = 5.18, *P* = 0.029; GSH: *F*_1,35_ = 0.00, *P* = 0.963; quality: *F*_1,35_ = 4.72, *P* = 0.037), but in this case the correlation between bib size and GSH levels in high-quality birds did not reach significance (*b* = 4.08, *t* = 1.85, *P* = 0.073; low-quality birds: *b* = −4.24, *t* = −1.52, *P* = 0.138). The interaction between treatment and quality was also significant in this model (*F*_1,35_ = 15.84, *P* < 0.001; treatment: *F*_1,35_ = 0.06, *P* = 0.809; tarsus length: *F*_1,35_ = 0.52, *P* = 0.474).

### EFFECTS OF BSO ON THE CHANGE IN CYSTEINE AND GSH LEVELS

**Figure 3:**
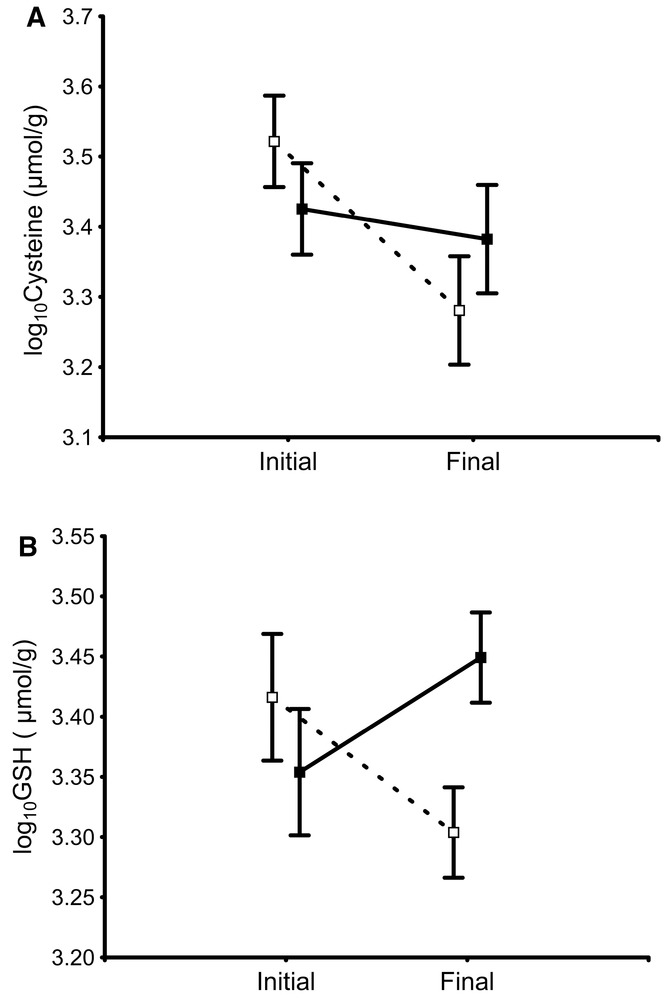
Change in the levels (least squares mean ± SE) of cysteine (A) and GSH (B) in erythrocytes of male house sparrows during the course of the experiment. Solid symbols and lines: control birds; open symbols and dashed lines: BSO-treated birds.

Neither the interaction between treatment and quality (*F*_1,42_ = 0.21, *P* = 0.646) nor quality alone (*F*_1,43_ = 0.00, *P* = 0.956) explained the change in cysteine levels. The final model only included a significant effect of treatment (*F*_1,46_ = 14.14, *P* < 0.001), which was due to a decrease in cysteine levels that occurred in BSO-treated birds (*P* < 0.0001) but not in controls (*P* = 0.255; Fig. 3A).

The final model for GSH included significant effects of treatment (*F*_1,37_ = 10.04, *P* = 0.003) and quality (*F*_1,37_ = 5.13, *P* = 0.029), but not the interaction between treatment and quality (*F*_1,36_ = 0.03, *P* = 0.866). The effect of treatment was due to the fact that GSH levels increased in controls (*P* = 0.047) but decreased in BSO-treated birds (*P* = 0.029), which made that final GSH levels were lower in BSO-treated birds than in controls (*P* = 0.032; Fig. 3B). The effect of quality was due to a tendency of high-quality birds to decrease GSH levels that was not observed in low-quality birds, although differences were not significant in any group (high-quality birds: *P* = 0.111, low-quality birds: *P* = 0.186).

### EFFECTS OF BSO ON MELANINS

**Figure 4:**
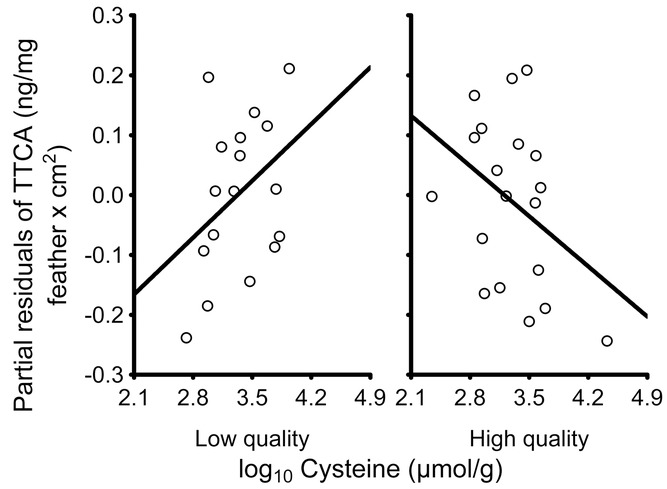
Relationship between final pheomelanin levels (TTCA levels multiplied by bib size) in bib feathers and cysteine levels in erythrocytes of low- and high-quality male house sparrows. The residual figures of the response variable are shown (i.e., partial effects after applying a GLM model without cysteine levels and quality). The lines are the regression lines.

Before analyzing the effects of BSO on the production of melanins, we analyzed the association between the levels of melanins and the levels of cysteine and GSH, as melanin production should depend on the latter, but the nature of the association may depend on the quality of birds. The final model for final levels of TTCA showed that this variable depended on the final levels of cysteine, but in interaction with the quality of birds (*F*_1,30_ = 6.42, *P* = 0.017; cysteine: *F*_1,30_ = 0.00, *P* = 0.956; quality: *F*_1,30_ = 6.72, *P* = 0.014). This interaction was due to a marginally significant negative correlation between TTCA and cysteine levels in high-quality birds (*b* = −0.14, *t* = −2.01, *P* = 0.053), whereas there was no correlation in low-quality birds (*b* = 0.15, *t* = 1.66, *P* = 0.107; Fig. 4). The other terms in the final model were treatment (*F*_1,30_ = 17.86, *P* < 0.001) and tarsus length (*F*_1,30_ = 0.85, *P* = 0.364). 4-AHP levels, by contrast, were not related to cysteine levels in interaction with quality (*F*_1,31_ = 3.89, *P* = 0.057; cysteine: *F*_1,31_ = 0.46, *P* = 0.504; quality: *F*_1,31_ = 4.21, *P* = 0.049). There was no association between TTCA and GSH levels (results not shown), but 4-AHP levels were positively related to GSH although not in interaction with quality (*b* = 0.05, *t* = 2.96, *F*_1,33_ = 8.74, *P* = 0.006; there were no other terms in the final model excepting tarsus length, *F*_1,33_ = 0.02, *P* = 0.875). Lastly, PTCA levels were not related to either cysteine or GSH levels (results not shown). Therefore, pheomelanin levels in feathers were negatively related to cysteine levels in erythrocytes as expected, but only in high-quality birds and independently of the experimental treatment.

**Figure 5:**
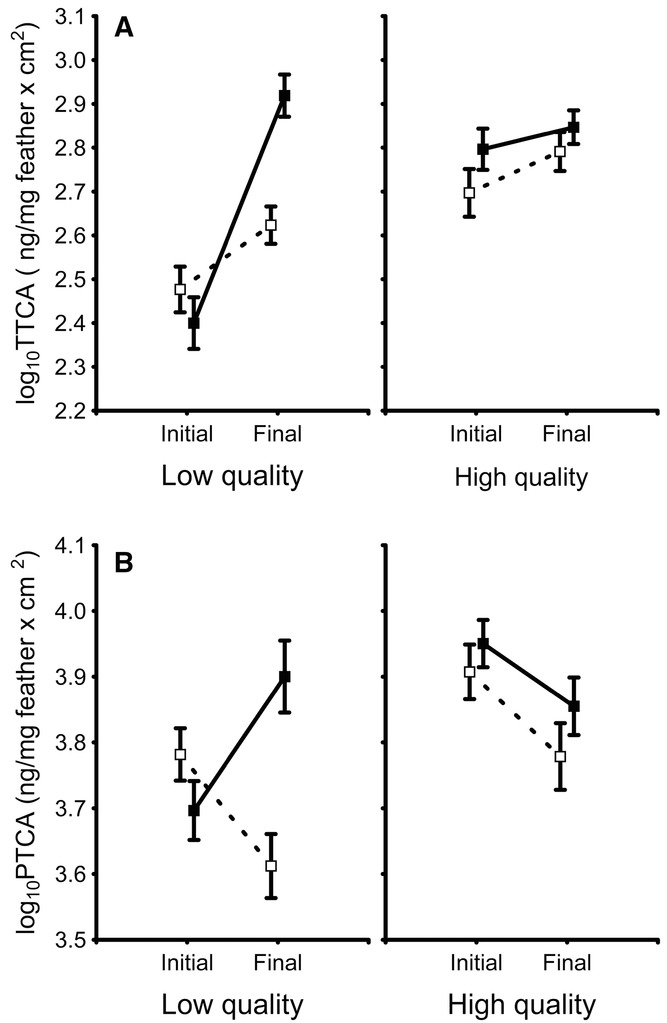
Change in pheomelanin levels (TTCA levels multiplied by bib size; A) and eumelanin levels (PTCA levels multiplied by bib size; B) in bib feathers of male house sparrows of low and high quality during the course of the experiment. Solid symbols and lines: control birds; open symbols and dashed lines: BSO-treated birds. Values are least squares mean ± SE.

When analyzing the effects of the experimental treatment on TTCA levels, the final model revealed a significant interaction between treatment and quality (*F*_1,34_ = 11.74, *P* = 0.002; treatment. *F*_1,34_ = 7.08, *P* = 0.012; quality: *F*_1,34_ = 18.76, *P* < 0.001; tarsus length: *F*_1,34_ = 0.96, *P* = 0.335) because, among low-quality birds, TTCA levels increased in both control (*P* < 0.0001) and BSO-treated birds (*P* = 0.012) but the increase was greater in the former so that final TTCA levels were higher in controls than in BSO-treated birds (*P* < 0.001; Fig. 5A). By contrast, there was no increase in TTCA levels in neither controls (*P* = 0.368) nor BSO-treated (*P* = 0.122) among high-quality birds (Fig. 5A). The final model for the other pheomelanin marker (4-AHP) did not result in any significant effect (results not shown). Thus, it is the pheomelanin marker that was related to bib size (i.e., TTCA) which was affected (lower increase relative to controls) by the experimental treatment, differentially regarding the quality of birds.

The final model for eumelanin (PTCA) levels revealed a similar significant interaction between treatment and quality (*F*_1,34_ = 8.40, *P* = 0.006; treatment: *F*_1,34_ = 11.67, *P* = 0.002; quality: *F*_1,34_ = 4.93, *P* = 0.033; tarsus length: *F*_1,34_ = 0.67, *P* = 0.420) because, among low-quality birds, PTCA levels increased in controls (*P* = 0.004) and decreased in BSO-treated birds (*P* = 0.007). Among high-quality birds, PTCA levels decreased significantly in BSO-treated birds (*P* = 0.043) and in a marginally nonsignificant manner in controls (*P* = 0.071; Fig. 5B). Thus, there was a differential variation in eumelanin levels regarding the quality of birds, with a strong decrease in birds treated with BSO as expected but an increase in controls among low-quality birds, whereas the same effects were less marked (BSO-treated) or even not observed (controls) among high-quality birds.

### EFFECTS OF BSO ON PLASMA ANTIOXIDANT CAPACITY

Neither treatment (*F*_1,41_ = 0.20, *P* = 0.658) nor quality (*F*_1,41_ = 0.58, *P* = 0.451) or their interaction (*F*_1,41_ = 0.79, *P* = 0.379) had a significant effect on the change in TAC levels in plasma after controlling for the change in uric acid levels (Wilks' *λ* = 0.18).

### EFFECTS OF BSO ON OXIDATIVE STRESS

**Figure 6:**
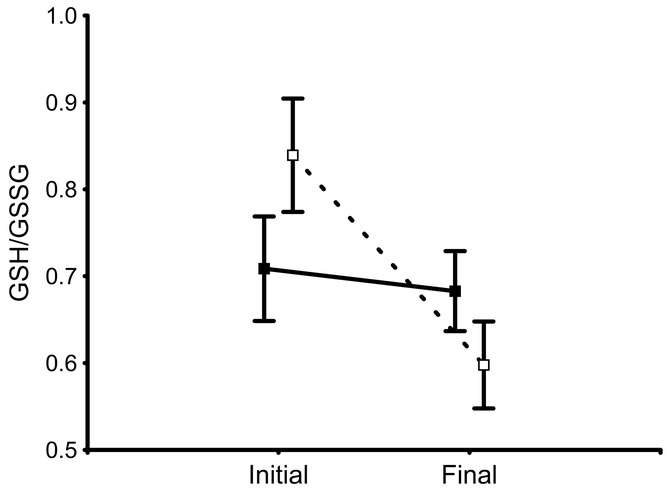
Change in the levels (least squares mean ± SE) of the ratio GSH/GSSG in male house sparrows of low (solid symbols and line) and high (open symbols and dashed line) quality during the course of the experiment.

The final model for the ratio GSH/GSSG only included bird quality, which was significantly related to the change in the ratio (*F*_1,35_ = 4.54, *P* = 0.040). The effect was due to a decrease in the ratio in birds of high quality (*P* = 0.002) that was not observed in birds of low quality (*P* = 0.708; Fig. 6). Thus, oxidative stress, as reflected by GSSG, increased independently of the experimental treatment in high-quality birds whereas low-quality birds avoided this increase.

By contrast, the change in MDA levels was not affected by neither treatment (*F*_1,43_ = 0.07, *P* = 0.791), quality (*F*_1,44_ = 0.55, *P* = 0.462) nor their interaction (*F*_1,42_ = 0.09, *P* = 0.771). The same was found for the change in body condition (treatment: *F*_1,45_ = 0.61, *P* = 0.439; quality: *F*_1,44_ = 0.39, *P* = 0.533; treatment × quality: *F*_1,43_ = 1.04, *P* = 0.314).

### EFFECTS OF BSO ON BIB EXPRESSION

**Figure 7:**
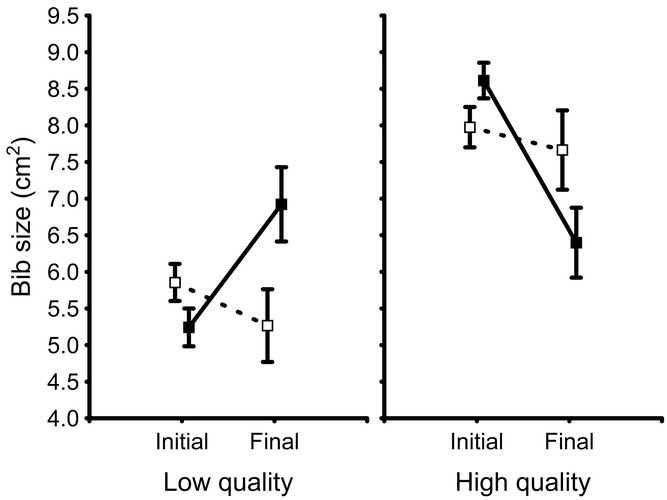
Change in bib size (least squares mean ± SE) of low- and high-quality male house sparrows during the course of the experiment. Solid symbols and lines: control birds; open symbols and dashed lines: BSO-treated birds.

The interaction between treatment and quality was significant at explaining the change in bib size (*F*_1,42_ = 15.05, *P* < 0.001; treatment: *F*_1,42_ = 0.12, *P* = 0.733; quality: *F*_1,42_ = 11.54, *P* = 0.001). Among low-quality birds, those treated with BSO did not change their bib size (*P* = 0.366) but avoided the “natural tendency” shown by control birds to increase their bib (*P* = 0.009; Fig. 7). Among high-quality birds, those treated with BSO neither change their bib size (*P* = 0.639) but avoided the “natural tendency” shown by control birds to decrease their bib (*P* < 0.001; Fig. 7). As a consequence, BSO only created phenotypes with larger bibs than controls in high-quality birds (*P* = 0.028), and by contrast the bibs of low-quality BSO-treated birds were smaller than the bibs of controls (*P* = 0.003; Fig. 7).

## Discussion

We succeeded in inducing the expression of better phenotypes (i.e., bibs larger than controls) with the treatment of BSO, but only in birds whose quality was high at the beginning of the experiment and not in those whose quality was low, where the resulting phenotype was actually worse than controls. However, when we analyzed the change in bib size among birds of the same treatment neither low-quality nor high-quality birds changed their bib size. Differences in final bib size between BSO-treated birds and controls arose because bib size increased in control low-quality birds and decreased in control high-quality birds.

This effect may be explained by the so-called regression to the mean, by which individuals tend to be closer to the mean in a second measurement of any variable (Kelly and Price 2005). In our case, however, results cannot be attributed to the regression to the mean effect for a simple reason: bib size only changed among control birds (Fig. 7). The impact of the social environment in this bird species may explain the finding. Our males were isolated in individual cages during molt and did not interact with other males. It is known that social environment affects bib size in male house sparrows, so that males that have higher rates of aggression during molt develop larger bibs than males that have lower aggression rates (McGraw et al. 2003). It is therefore likely that, under equal rates of aggressive interactions as those to which our birds were exposed during molt, all control birds tended to develop average size bibs regardless of their quality. This tendency was only absent in birds exposed to the experimental treatment, in which the effect of BSO led to an avoidance of the tendency observed in control birds of the respective quality groups. Therefore, in each quality group, control birds showed a “natural” tendency about bib expression (increase in low-quality birds and decrease in high-quality birds). BSO-treated birds avoided this inertia by not changing their bib size during molt, and we believe that the mechanisms by which this avoidance was achieved may be informative about the honesty of bib size, as it was only through this avoidance by which BSO induced the expression of better phenotypes in high-quality birds. We discuss these mechanisms below.

Final bib size was negatively correlated with TTCA levels in all birds (Galván et al. 2014a), and negatively correlated with cysteine levels in high-quality birds only, whereas it was not significantly correlated with GSH levels in any group of birds. On the basis of these three associations, we can estimate how the changes in cysteine, GSH, and TTCA levels affected bib size during the course of the experiment. We assumed that bib size was at least partly explained by cysteine (and GSH) levels and partly by TTCA levels. These predictions are shown in Table 1. We expected that cysteine levels would decrease as a consequence of the increase in cysteine catabolism that BSO induces (Griffith 1982), and cysteine levels accordingly decreased in both low- and high-quality birds treated with BSO. This decrease in cysteine levels should positively affect bib size in high-quality birds but not in low-quality birds, as final bib size was negatively correlated with cysteine levels in high-quality birds only (Table 1). The inhibitory effect of BSO on γ-glutamylcysteine synthetase should also lead to a decrease in GSH levels (Griffith 1982; Dizdar et al. 1997; Galván and Alonso-Alvarez 2008), and this expectation was also fulfilled in both low- and high-quality birds treated with BSO. However, this decrease in GSH levels should not have a strong effect on bib size as derived from a weak correlation between final bib size and GSH levels. BSO should have a negative but weak effect on pheomelanin production (Galván et al. 2014b), but we found that control low-quality birds showed a stronger increase in TTCA levels compared to other groups. Although the increase among low-quality birds was more marked in controls than in birds treated with BSO, TTCA levels also increased in the latter so the change in TTCA levels was not qualitatively different (both increased) in control and BSO-treated birds, and the lack of change among high-quality birds was also common for control and BSO-treated birds. This suggests that the change in TTCA levels during molt was not due to the experimental treatment, as derived from the weak effects on pheomelanin production found in an in vitro experiment (Galván et al. 2014b). Changes in TTCA levels, although not mediated by the experimental treatment, should have a negative effect on bib size in low-quality birds and no effect in high-quality birds, given the negative association between final bib size and TTCA levels (Table 1). Lastly, the natural tendency to increase bib size in control low-quality birds should exert a positive effect on bib development in BSO-treated low-quality birds, whereas the natural tendency to decrease bib size in high-quality control birds should exert a negative effect on bib development in BSO-treated high-quality birds (Table 1). A simple rule assigning different values to these predictions shows that there is congruence with predictions based on these physiological parameters and the observed results indicating a lack of change in bib size during molt in both low- and high-quality birds treated with BSO (Table 1).

**Table 1:** Summary of the observed effects of the experimental treatment with BSO on the levels of cysteine, GSH, and pheomelanin (TTCA) and on bib size in low- and high-quality male house sparrows

	Low-quality birds		High-quality birds	
	Observed BSO effect	Predicted result of BSO effect on bib size	Observed BSO effect	Predicted result of BSO effect on bib size
Cysteine	Decrease	No effect (0)	Decrease	Increase (+1)
GSH	Decrease	No effect (0)	Decrease	No effect (0)
TTCA	Increase	Decrease (−1)	No effect	No effect (0)
Natural tendency in bib size change		Increase (+1)		Decrease (−1)
Result on bib size	No effect	0 + 0 −1 +1 = 0 (no effect)	No effect	+1 + 0 + 0 −1 = 0 (no effect)
Congruence between observation and prediction	Yes		Yes	

The predicted results that these effects should have on the bib size of birds are also depicted, assigning a value of −1 to predicted negative results, 0 to neutral results, and +1 to positive results. Predicted results are based on the observed associations between final bib size and the levels of cysteine, GSH, and TTCA. The natural tendency in bib size change, determined as the observed change in the bib size of control birds, is included as a predicted influence on bib expression in BSO-treated birds. Note that what prevents low-quality birds from having a positive result on bib size is the lack of predicted effect of the experimental decrease in cysteine levels on bib size, contrary to high-quality birds.

In low-quality birds treated with BSO, final bib size was not associated with cysteine levels, which made that their bib did not increase during molt despite being exposed to a reduction in their cysteine levels. Indeed, only the bib size of high-quality birds was sensitive to variation in cysteine levels, and accordingly, pheomelanin levels were negatively associated with cysteine levels in high-quality birds but the pattern was much less marked in low-quality birds treated with BSO (Fig. 4). This negative relationship between pheomelanin and cysteine levels is expected from the fact that pheomelanin production consumes cysteine, as the latter reacts with dopaquinone to form 5-*S*-cysteinyldopa, the main intermediate of the monomeric subunits for pheomelanin (García-Borrón and Olivares Sánchez 2011; Ito et al. 2011; Pavel et al. 2011). This known cause-effect relationship between pheomelanin and cysteine levels also means that, as cysteine serves as a substrate for pheomelanin production, decreases in cysteine levels should lead to a decrease in pheomelanin levels despite a negative correlation is observed between both variables (arisen as a consequence of cysteine consumption during pheomelanin production). The normal, expected mechanism by which pheomelanin is produced, and on which bib expression depends, is thus observed in high-quality birds but not in low-quality birds. We propose that this capacity of low-quality BSO birds to avoid that a decrease in cysteine levels leads to the development of larger bibs is mediated by a mechanism that may have evolved to avoid the production of traits whose level of expression does not correspond to the quality of the bearers. The lack of association between pheomelanin and cysteine levels may have also allowed low-quality birds to avoid an increase in oxidative stress, as we actually found, because the lack of association means that cysteine (i.e., an antioxidant resource, as cysteine is a constitutive amino acid of GSH, the main intracellular antioxidant; Lu 1999) is not used at a large extent for pheomelanin production, and more cysteine is therefore available for antioxidant protection (Galván and Møller 2011; Galván et al. 2011, 2012).

Initially, we predicted that the experimental induction of larger bibs would affect to both high- and low-quality birds but the latter would suffer from greater oxidative stress levels if it is production costs what prevents low-quality birds from producing large bibs as assumed by the immunocompetence handicap hypothesis (González et al. 1999; Buchanan et al. 2003; Laucht et al. 2011; Laucht and Dale 2012). Alternatively, we proposed that if some physiological mechanism avoids dishonesty in signal size, the experimental induction of larger bibs would be only observed in high-quality birds but blocked in low-quality birds. Our study is the first in directly manipulating the constitutive pigment of the bib of male house sparrows. The results support the last but not the first prediction. We created the physiological conditions favoring the expression of large bibs (i.e., reduced levels of cysteine that should lead to low pheomelanin levels that in turn should lead to large bibs) in all birds treated with BSO, but this resulted in the induction of better phenotypes in high-quality birds only. As a consequence, low-quality birds did not express the phenotype disproportionately regarding their intrinsic quality. This could have been made by blocking the mechanism by which cysteine is used to produce pheomelanin, their bib expression thus being insensitive to variations in cysteine levels. We suggest that this mechanism is the core of the honesty in the bib size of male house sparrows: An uncoupling between cysteine availability and pheomelanin production seems to have evolved in low-quality birds so that, if cysteine levels decrease as a consequence of endogenous or exogenous factors, pheomelanin production is not reduced and the development of large bibs is not induced. This may be possible for low-quality birds by establishing a constant level of cysteine assigned for pheomelanin production, so that variations in cysteine levels do not affect pheomelanin production as long as total cysteine levels are above the threshold. High-quality birds, by contrast, may produce pheomelanin proportionally to cysteine levels, thus being sensitive to variations in the latter.

In the recent years, it has become evident that costs that should make the production of large signals unaffordable to low-quality signalers are not easy to find (Zollman et al. 2013), and that identifying the mechanisms that prevent low-quality signalers from sending the costly signal is key to understand how signal honesty is maintained (Lachmann et al. 2001; Számadó 2011; Holman 2012). Our results agree with these assertions and suggest for the first time that greater physiological costs are not found in low-quality signalers because mechanisms may have evolved to avoid the production of disproportionately large signals even if physiological conditions are favoring it. Indeed, we found that oxidative stress as reflected by the ratio GSH/GSSG seemed to increase during the course of the experiment in male house sparrows of high-quality independently of the treatment, perhaps as a consequence of the maintenance of larger bibs on average, which implies maintaining low levels of the antioxidant resource cysteine. This cost in antioxidant terms may not be affordable by low-quality birds, which in case of not having evolved the mechanism by which pheomelanin production is uncoupled from cysteine levels, may have faced a greater oxidative stress as predicted by the handicap principle (Zahavi 1975; Grafen 1990). Therefore, differential production and maintenance costs may keep the honesty of costly signals, but physiological mechanisms have probably evolved to avoid the production of bluffs in low-quality signalers and prevent them from facing greater costs when environmental influences (e.g., stressful social interactions, McGraw et al. 2003) lead to physiological conditions favoring the production of large signals. We suggest that the uncoupling between pheomelanin and cysteine may constitute one such mechanism for melanin-based signals of quality.

## DATA ARCHIVING

The doi for our data is 10.5061/dryad.71mh5.
